# Tissue-specific expression analysis of Na^+^ and Cl^−^ transporter genes associated with salt removal ability in rice leaf sheath

**DOI:** 10.1186/s12870-020-02718-4

**Published:** 2020-11-03

**Authors:** Sarin Neang, Itsuki Goto, Nicola Stephanie Skoulding, Joyce A. Cartagena, Mana Kano-Nakata, Akira Yamauchi, Shiro Mitsuya

**Affiliations:** 1 Graduate School of Bioagricultural Sciences, Nagoya University, Chikusa, Nagoya, 464-8601 Japan; 2 Graduate School of Science, Nagoya University, Chikusa, Nagoya, 464-8601 Japan; 3 International Center for Research and Education in Agriculture, Nagoya University, Chikusa, Nagoya, 464-8601 Japan

**Keywords:** Salt tolerance, Leaf sheath, Salt removal ability, Fundamental parenchyma cells, Na^+^ and Cl^−^ transporters

## Abstract

**Background:**

A significant mechanism of salt-tolerance in rice is the ability to remove Na^+^ and Cl^−^ in the leaf sheath, which limits the entry of these toxic ions into the leaf blade. The leaf sheath removes Na^+^ mainly in the basal parts, and Cl^−^ mainly in the apical parts. These ions are unloaded from the xylem vessels in the peripheral part and sequestered into the fundamental parenchyma cells at the central part of the leaf sheath.

**Results:**

This study aimed to identify associated Na^+^ and Cl^−^ transporter genes with this salt removal ability in the leaf sheath of rice variety FL 478. From 21 known candidate Na^+^ and Cl^−^ transporter rice genes, we determined the salt responsiveness of the expression of these genes in the basal and apical parts, where Na^+^ or Cl^−^ ions were highly accumulated under salinity. We also compared the expression levels of these transporter genes between the peripheral and central parts of leaf sheaths. The expression of 8 Na^+^ transporter genes and 3 Cl^−^ transporter genes was up-regulated in the basal and apical parts of leaf sheaths under salinity. Within these genes, *OsHKT1;5* and *OsSLAH1* were expressed highly in the peripheral part, indicating the involvement of these genes in Na^+^ and Cl^−^ unloading from xylem vessels. *OsNHX2, OsNHX3, OsNPF2.4* were expressed highly in the central part, which suggests that these genes may function in sequestration of Na^+^ and Cl^−^ in fundamental parenchyma cells in the central part of leaf sheaths under salinity. Furthermore, high expression levels of 4 candidate genes under salinity were associated with the genotypic variation of salt removal ability in the leaf sheath.

**Conclusions:**

These results indicate that the salt removal ability in rice leaf sheath may be regulated by expressing various Na^+^ or Cl^−^ transporter genes tissue-specifically in peripheral and central parts. Moreover, some genes were identified as candidates whose expression levels were associated with the genotypic variation of salt removal ability in the leaf sheath. These findings will enhance the understanding of the molecular mechanism of salt removal ability in rice leaf sheath, which is useful for breeding salt-tolerant rice varieties.

**Supplementary Information:**

The online version contains supplementary material available at 10.1186/s12870-020-02718-4.

## Background

The rice leaf sheath has been found to play an important role in decreasing toxic ion such as Na^+^ and Cl^−^ concentrations in the leaf blade, the major photosynthetic tissue of rice plants [1–3]. The salt removal ability in leaf sheaths consists of salt unloading from xylem and salt sequestration into the leaf sheath cells, which is done by two different inner parts, peripheral and central parts in coordination with each other. The leaf sheath unloads Na^+^ and Cl^−^ ions from xylem vessels in the vasculature in the peripheral part, then preferentially transports these ions from the peripheral part to the central part, and sequestering those ions into the fundamental parenchyma cells in the central part [3]. Additionally, Na^+^ and Cl^−^ were accumulated into different regions of the leaf sheath, Na^+^ in basal part and Cl^−^ in apical part, suggesting that responsive genes for the transport of those ions might be up-regulated in each of these locations in the rice leaf sheath [3]. Therefore, to elucidate the molecular mechanism of salt removal ability in the leaf sheath, it is important to identify which known candidate Na^+^ and Cl^−^ transporter genes are associated with the removal of Na^+^ in the basal part and Cl^−^ in the apical of leaf sheaths, and with salt unloading in the peripheral part and salt sequestration in the central fundamental parenchyma cells, under salinity.

Previously, Na^+^ unloading from the xylem vessels has been reported to be mediated by several group 1 high-affinity K^+^ transporter (HKT1) proteins [4–6]. AtHKT1;1 in *Arabidopsis* [7–9] and OsHKT1;1 in rice [10] function in Na^+^ unloading from the xylem vessels in roots, which contributes to salt tolerance. A number of studies suggested that the *OsHKT1;4* gene in rice mediates Na^+^ exclusion in the leaf sheath to prevent over-accumulation of Na^+^ in the leaf blade, especially at the reproductive stage [2, 11, 12]. Moreover, another group 1 HKT transporter, *OsHKT1;5*, has been known to be preferentially expressed in root parenchyma cells close to the xylem vessels and physiologically functions in retrieving Na^+^ from the xylem sap, resulting in less Na^+^ concentration and increase of K^+^ level in shoot under salt stress [13–15]. At the leaf sheath level, the *OsHKT1;5* gene is also responsible for unloading Na^+^ from xylem vessels into xylem parenchyma cells [16]. *HKT2* genes encoding class 2 high-affinity K^+^ transporter proteins also mediate inward uptake of Na^+^ as well as K^+^ [17–19].

Other important Na^+^-selective transporter genes known as tonoplast-localized Na^+^/H^+^ exchanger (NHX), are responsible for the sequestration of Na^+^ into the vacuole, leading to the reduction of toxic Na^+^ in the cytosol, in several plant species during salt stress [20–25]. Overexpression of *AtNHX1* in *Arabidopsis* increases salt tolerance by compartmentalizing Na^+^ into the vacuole in response to salt stress [20, 21]. In rice, *OsNHX1, OsNHX2, OsNHX3* and *OsNHX5* expresses in various parts such as panicles, flag leaf sheath and blade, seedling shoots and roots, efficiently functions in Na^+^ sequestration in the vacuole to maintain low Na^+^ in the cytosol during salt stress, which is essential for salt tolerance in rice [22, 24].

Furthermore, plasma membrane-type Na^+^/H^+^ exchanger protein Salt-Overly-Sensitive 1 (SOS1) is one of important Na^+^ transporters to reduce the transfer of Na^+^ from roots to shoots in *Arabidopsis*, rice and bread wheat [26–30]. SOS1 has been reported to function in both Na^+^ extrusion from the root epidermis [5, 31] and Na^+^ loading into the xylem in roots and shoots [26, 32–34]. Wu et al. [29] revealed that Na^+^ extrusion from the root elongation zone mediated by *TaSOS1* is essential for salt tolerance in wheat. In rice, a recent study by Mahi et al. [34] reported that *OsSOS1* plays important roles to enhance salt tolerance in rice by mediating Na^+^ exclusion and loading Na^+^ into the xylem in rice plants.

Several previous studies have illustrated some transporter genes associated with mechanism of Cl^−^ transport and detoxification under salt stress in plants. In *Arabidopsis*, Nitrate Transporter 1/Peptide Transporter 2.4 (NPF2.4) and Slow-Type Anion Channel–Associated Homolog 1 (SLAH1) proteins, localized at the plasma membrane in root stele cells, mediate Cl^−^ loading into the xylem vessels and control long-distance transport of Cl^−^ from roots to shoots [35, 36]. Moreover, cation-chloride cotransporter 1 (CCC1), which is also localized at the plasma membrane of root stele cells and leaves, is involved in Cl^−^ unloading from the xylem vessels to the surrounding parenchyma cells, affecting long-distance Cl^−^ transport in *Arabidopsis* and rice under salt stress [37, 38]. Additionally, Nakamura et al. [39] showed that the tonoplast-localized OsCLC1 and OsCLC2 proteins, belonging to the chloride channel (CLC) family, function in compartmentalizing Cl^−^ into the vacuole to avoid toxicity of Cl^−^ in the cytosol in rice under saline conditions.

In this study, we aimed to determine which known candidate Na^+^ or Cl^−^ transporter genes are associated with the salt removal ability in leaf sheaths of rice via general and localized expression analysis. Firstly, we determined the transcript levels of candidate Na^+^ and Cl^−^ transporter genes in the basal (Na^+^) and apical (Cl^−^) parts of the leaf sheath in the rice genotype FL 478, respectively, where Na^+^ and Cl^−^ were accumulated at the highest level [3] to identify the genes whose expression levels increase in response to salt stress. Secondly, we compared the transcript levels of candidate Na^+^ and Cl^−^ transporter genes between the peripheral and central regions of the leaf sheath of FL 478 plants grown under saline conditions. The peripheral part includes vasculatures, epidermis and peripheral fundamental parenchyma cells [3]. We hypothesized that Na^+^ or Cl^−^ transporter genes that are highly expressed in the peripheral part may be involved in unloading of these ions in vasculature, or accumulation in the peripheral tissues. On the other hand, because the central part mostly includes fundamental parenchyma cells where salt is highly accumulated [3], Na^+^ or Cl^−^ transporter genes expressed highly in the central part were hypothesized to be involved in sequestration of these ions into fundamental parenchyma cells. To accomplish these objectives, we selected key Na^+^ and Cl^−^ transporter genes (Table 1) that have been previously described to be related to enhancing salt tolerance in rice. Then, the expression analysis of all candidate genes was performed in the basal, apical part and internal tissues of rice leaf sheaths under normal and NaCl-treated conditions. Next, to identify what gene expression profile is associated with the genotypic difference in the salt removal ability in the leaf sheath, we chose two pairs of two rice genotypes that showed superior or inferior Na^+^ or Cl^−^ removal ability in the leaf sheath [40] and compared the expression levels of candidate Na^+^ or Cl^−^ transporter genes in the leaf sheath tissues under salinity. Finally, we discussed potential candidate transporter genes involved in the salt removal ability in leaf sheaths of rice.

**Table 1:** All candidate genes and the physiological functions used in this study

Ion	Transporter gene	Candidate gene	Function	Reference
Na^+^	**HKT** High-affinity K^+^ transport (Plasma membrane)	*OsHKT1;1* *OsHKT1;3* *OsHKT1;4* *OsHKT1;5* *OsHKT2;1* *OsHKT2;3* *OsHKT2;4*	Control root-to-shoot transfer of Na^+^ by unloading of Na^+^ from the xylem into xylem parenchyma cells, and reduce Na^+^ and increasing K^+^ levels in shoots during salt stress	[2, 7, 10–13, 18, 19]
	**NHX** Na^+^/H^+^ Exchanger (Tonoplast)	*OsNHX1* *OsNHX2* *OsNHX3* *OsNHX4* *OsNHX5*	Maintain low Na^+^ in the cytosol under salt stress by vacuolar Na^+^ sequestration	[24]
	**SOS** Salt Overly Sensitive (Plasma membrane)	*OsSOS1*	Mediate Na^+^ loading into the xylem	[27, 34]
Cl^−^	**NPF** Nitrate Transporter 1/Peptide Trasnport Family (Plasma membrane)	*OsNPF2;4*	Mediate Cl^−^ loading to the root xylem	[35]
	**CLC** Chloride Channel (Tonoplast)	*OsCLC1* *OsCLC2* *OsCLC5* *OsCLC6*	Function in compartmentalizing Cl^−^ ions into the vacuole in rice	[39]
	**CCC** Cation-Chloride Cotransporter (Plasma membrane)	*OsCCC1*	Mediate Cl^−^ loading and unloading between xylem parenchyma cells and xylem vessels	[38]
	**SLAH** Slow-Type Anion Channel-Associated Homolog (Plasma membrane)	*OsSLAH1* *OsSLAH7*	Mediate Cl^−^ loading to the root xylem	[36]

## Results

### Distribution of Na^+^ and Cl^−^ in rice leaf

**Fig. 1:**
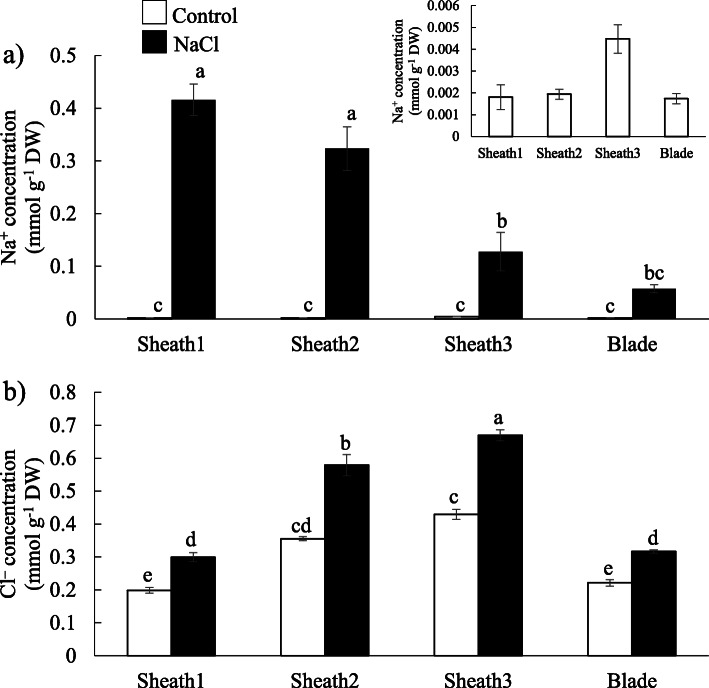
Na^+^ and Cl^−^ concentrations along the longitudinal axis of 5th leaves of FL 478 under control or treatment conditions with 100 mM NaCl. Sheath1 to Sheath3, from the basal to upper parts of leaf sheaths; Blade, whole leaf blade. Data are mean of three replications ± the standard error. Different letters indicate significant differences at *P* < 0.05 (Tukey’s multiple comparison test)

The distribution of Na^+^ and Cl^−^ along the longitudinal axis of the 5th leaves was determined using the salt-tolerant rice variety FL 478 under control and NaCl-treated conditions. Under NaCl treatment for 72 h, Na^+^ was accumulated higher in the basal part (Sheath1) of leaf sheaths and decreased towards the apical part of leaf blades (Fig. 1a). Under control conditions, Na^+^ concentration was relatively high in the apical parts of leaf sheaths, although there was no significant difference between each individual part (Fig. 1a). The Cl^−^ concentration was higher in the apical part of leaf sheath compared with other parts under both control and NaCl treatment (Fig. 1b).

### Expression profiles of Na^+^ and Cl^−^ transporter genes in the basal and apical parts of leaf sheaths

**Fig. 2:**
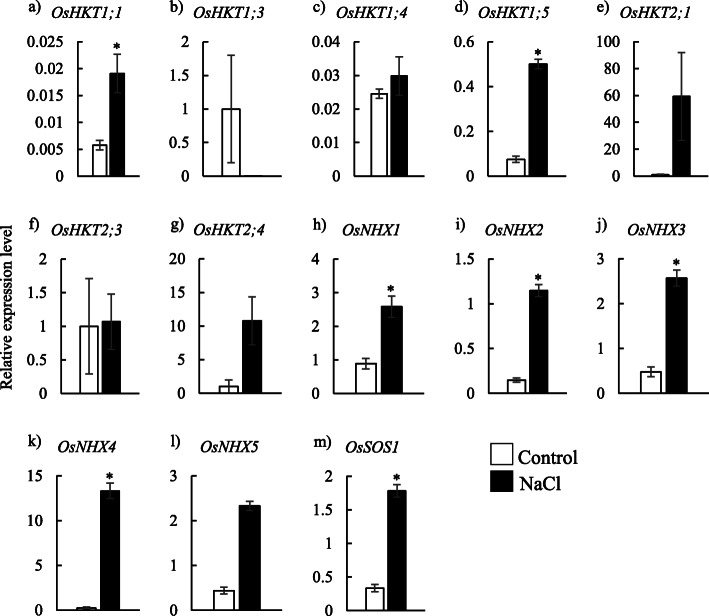
Relative expression levels of Na^+^ transporter genes in the basal part of leaf sheaths under control or treatment conditions with 100 mM NaCl. Data are mean of three replications ± the standard error. * indicates significant difference at *P* < 0.05 between conditions

Transcriptional expression analysis was conducted to determine the expression profiles of each Na^+^ and Cl^−^ transporter genes in the basal and apical parts of leaf sheaths, where Na^+^ or Cl^−^ was highly accumulated, under control or NaCl-treated conditions. Among all known Na^+^ transporter genes used in this study (13 in total), *OsHKT1;1*, *OsHKT1;5*, *OsSOS1*, *OsNHX1*, *OsNHX2*, *OsNHX3*, *OsNHX4* and *OsNHX5* showed a significant increase in their expression levels in the basal part of leaf sheath in response to NaCl treatment (Fig. 2).

**Fig. 3:**
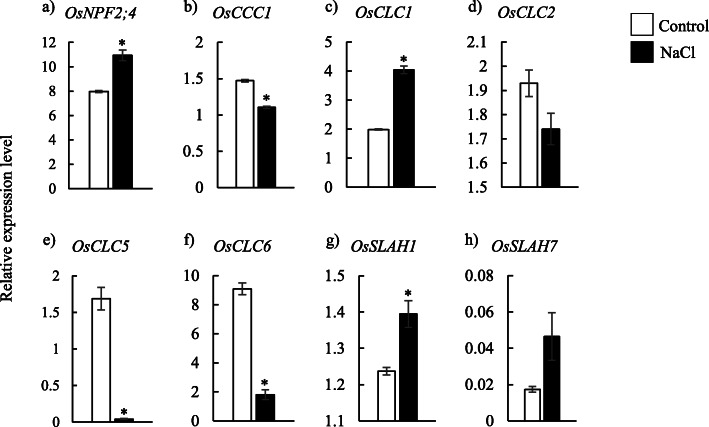
Relative expression levels of Cl^−^ transporter genes in the apical part of leaf sheath under control or treatment conditions with 100 mM NaCl. Data are mean of three replications ± the standard error. * indicates significant difference at *P* < 0.05 between conditions

Within known Cl^−^ transporter genes (8 in total), *OsNPF2;4*, *OsCLC1* and *OsSLAH1* were significantly up-regulated in the apical part of leaf sheaths under saline conditions (Fig. 3). The expression of *OsCCC1, OsCLC5* and *OsCLC6* were decreased in the apical part of leaf sheaths under NaCl treatment (Fig. 3).

The expression analysis of Na^+^ transporter genes was also conducted in the middle and apical parts of the leaf sheath (Additional file 1). In the middle part of the leaf sheath, only *OsNHX2,* among all Na^+^ transporter genes, showed a significant increase of expression level in response to salt stress (Additional file 1). Regarding the apical parts of leaf sheath, only *OsHKT1;4* showed a significant increase of expression level when exposed to salt stress (Additional file 1). The expression levels of all Cl^**−**^ transporter genes were also determined in the basal and middle parts of the leaf sheath (Additional file 2). In the basal parts of the leaf sheath, the expression levels of *OsNPF2;4*, *OsCCC1*, *OsCLC1*, *OsCLC2*, *OsSLAH1* and *OsSLAH7* significantly increased under salinity (Additional file 2). For the middle parts of leaf sheath, *OsCLC1* and *OsSLAH1* showed a significant increase in response to salt stress (Additional file 2).

### Expression profiles of Na^+^ transporter genes in the internal tissues of leaf sheaths

**Fig. 4:**
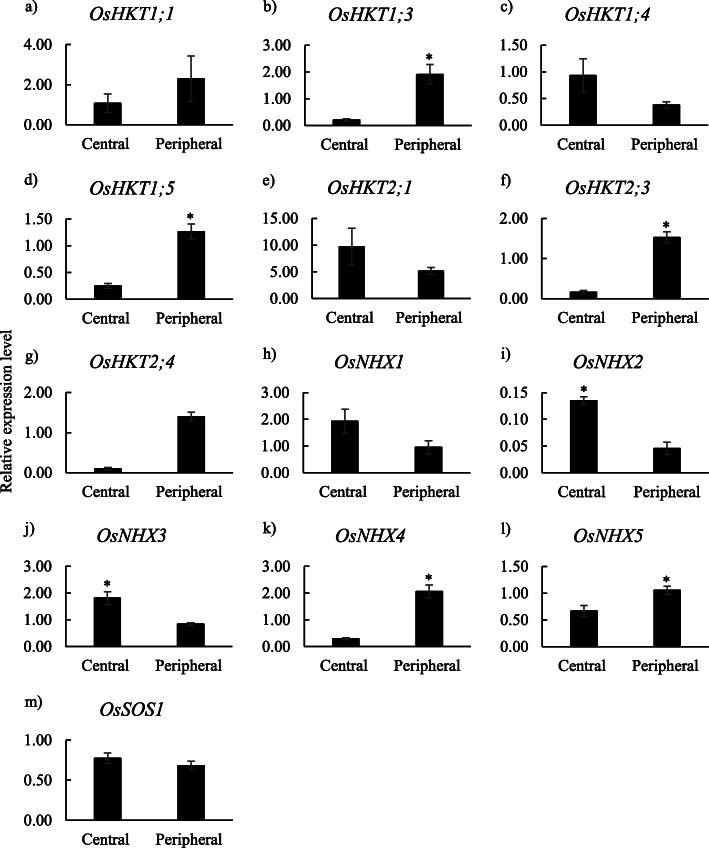
Relative expression levels of Na^+^ transporter genes in the central and peripheral parts of leaf sheath under treatment conditions with 100 mM NaCl. Data are mean of three replications ± the standard error. * indicates significant difference at *P* < 0.05 between two parts

Expression analysis of Na^+^ transporter genes in the internal tissues of leaf sheaths showed that peripheral parts had higher expression of *OsHKT1;3*, *OsHKT1;5*, *OsHKT2;3*, *OsHKT2;4*, *OsNHX4* and *OsNHX5* compared with the central parts under NaCl-treated conditions (Fig. 4). On the other hand, *OsNHX2 and OsNHX3* expression levels were higher in the central parts in comparison to the peripheral parts under salt stress (Fig. 4).

Under control conditions, the peripheral parts had higher expression levels of *OsHKT1;3*, *OsHKT1;5*, *OsHKT2;3*, *OsHKT12;4 and OsNHX5* than the central parts (Additional file 3). In contrast, *OsHKT1;4*, *OsHKT2;1* and *OsNHX2* expression levels were higher in the central parts in comparison to the peripheral parts (Additional file 3).

### Expression profiles of Cl^−^ transporter genes in the internal tissues of leaf sheaths

**Fig. 5:**
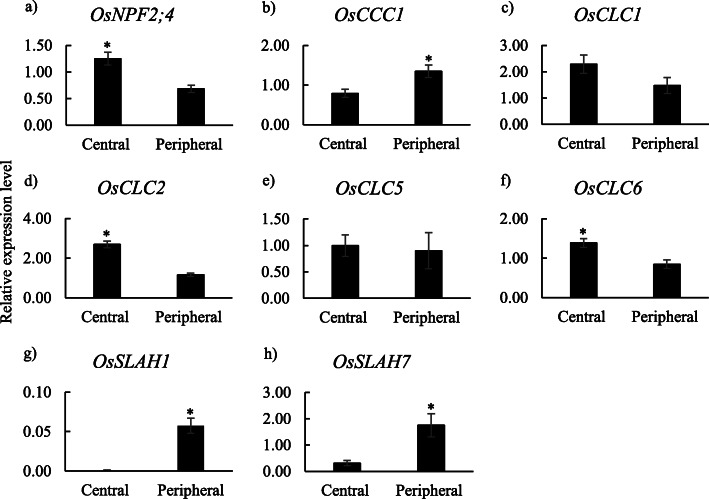
Relative expression levels of Cl^−^ transporter genes in the central and peripheral parts of leaf sheath under treatment conditions with 100 mM NaCl. Data are mean of three replications ± the standard error. * indicates significant difference at *P* < 0.05 between two parts

For the expression analysis of Cl^−^ transporter genes, *OsCCC1, OsSLAH1* and *OsSLAH7* had significantly higher expression levels in the peripheral parts compared with the central parts under NaCl-treated conditions (Fig. 5). The expression levels of *OsNPF2;4*, *OsCLC2* and *OsCLC6* were higher in the central parts under NaCl-treated conditions compared with the peripheral part (Fig. 5).

Under control conditions, *OsSLAH1* and *OsSLAH7* showed higher expression levels in the peripheral parts compared with the central parts (Additional file 4). On the other hand, no gene showed higher expression levels in the central part under control conditions (Additional file 4).

### Validations of candidate Na^+^ and Cl^−^ transporter genes using RNA-seq

RNA-seq analysis was performed to validate the results of the Real-Time PCR analysis on the expression levels of Na^+^ and Cl^−^ transporter genes in the central and peripheral parts of leaf sheath under normal and saline conditions. Under NaCl-treated conditions, the results of RNA-seq (Additional files 5, 6) showed comparable trends with the difference in the expression levels between peripheral and central parts as shown in the results of Real-Time PCR for all Na^+^ and Cl^−^ transporter genes except *OsCLC1* (Figs. 4, 5). Regarding *OsCLC1*, no available data of *OsCLC1* was obtained in RNA-seq since there is no RAP ID which was used for RNA-seq analysis, registered for this gene. The RNA-seq results showed that the peripheral parts had relatively higher expression levels of Na^+^ transporter genes such as *OsHKT1;3*, *OsHKT1;5*, *OsHKT2;3* and *OsHKT2;4* and a Cl^−^ transporter gene, *OsSLAH1*, compared with the central parts under NaCl-treated conditions (Additional files 5, 6). The central parts had relatively high expression levels of Na^+^ transporter genes such as *OsHKT1;4*, *OsHKT2;1* and *OsNHX3* and a Cl^−^ transporter gene, *OsCLC2*, in comparison to the peripheral parts under salinity (Additional files 5, 6).

Under normal conditions, the trends in the difference in the expression levels between peripheral and central parts were comparable between the results of RNA-seq and Real-Time PCR analyses for all Na^+^ and Cl^−^ transporter genes except *OsNHX2* (Additional files 3, 4, 7, 8). In RNA-seq analysis, the peripheral parts had relatively higher expression levels of *OsHKT1;3*, *OsHKT2;3*, *OsHKT12;4 and OsNHX2* for Na^+^ transporter genes and *OsSLAH1* for Cl^−^ transporter genes than the central parts under control conditions (Additional files 7, 8). In addition, among Na^+^ and Cl^−^ transporter genes, only the *OsHKT2;1* expression level was relatively higher in the central parts in comparison to the peripheral parts under normal conditions (Additional files 7, 8).

### Genotypic comparison of Na^+^ and Cl^−^ transporter gene expressions

**Fig. 6:**
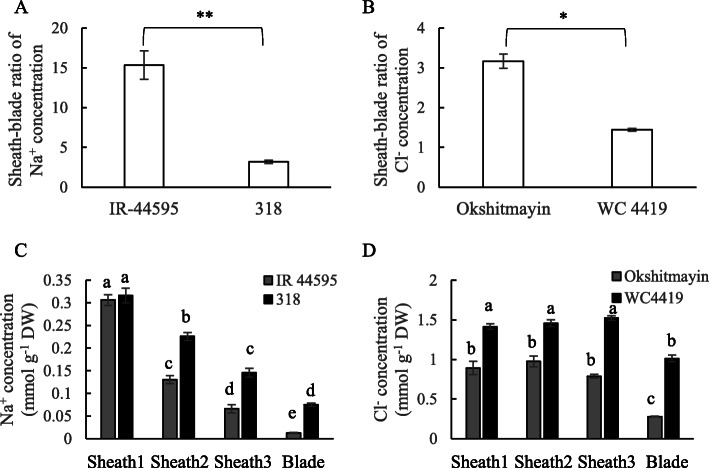
Genotypic comparison of sheath-blade ratio of Na^+^ and Cl^−^ concentrations and the accumulation pattern along the longitudinal axis of 5th leaves under salinity. The sheath-blade ratio of Na^+^ (**a**) and Cl^−^ (**b**) concentration in the 5th leaves between IR-44595 and 318 (for Na^+^) and Okshitmayin and WC 4419 (for Cl^−^). **c**, **d** Sheath1 to Sheath3, from the basal to upper parts of leaf sheaths; Blade, whole leaf blade. Data are mean of three replications ± the standard error (*n* = 3). * and ** indicate significant difference at *P* < 0.05 and 0.01 between two rice genotypes. Different letters indicate significant differences at *P* < 0.05 (Tukey’s multiple comparison test)

We compared the gene expression levels of Na^+^ and Cl^−^ transporter genes in the leaf sheath under salinity between IR-44595 and 318 for Na^+^ and Okshitmayin and WC 4419 for Cl^−^ that showed contrasting ability of Na^+^ or Cl^−^ removal ability in leaf sheath [40]. As reported previously [40], IR-44595 showed a higher sheath-blade ratio of Na^+^ concentration than 318 (Fig. 6a). Also, Okshitmayin showed a higher sheath-blade ratio of Cl^−^ concentration compared with WC 4419 (Fig. 6b).

IR-44595 and 318 accumulated the highest Na^+^ concentration in the basal leaf sheath under salinity, then the Na^+^ concentration decreased toward the leaf blade (Fig. 6c). On the other hand, Okshitmayin and WC 4419 showed a comparable Cl^−^ concentration among basal, middle, and apical parts of the leaf sheath (Fig. 6d). Therefore, for gene expression analysis using the active salt removal parts, we used the basal part of leaf sheaths for IR-44595 and 318, and the apical part of leaf sheaths for Okshitmayin and WC 4419.

Table 2 shows a comparison of the relative expression levels of Na^+^ transporter genes in the basal leaf sheaths of IR-44595 and 318 under salinity. Within the determined genes, the relative expression levels of *OsHKT1;3*, *OsHKT1;5* and *OsNHX1* were significantly higher in IR-44595 in comparison to 318. On the other hand, when compared to the relative expression levels of Cl^−^ transporter genes under salinity, the relative expression level of *OsCLC2* was significantly higher in Okshitmayin compared with WC 4419 (Table 3). *OsNHX4*, *OsSLAH1* and *OsSLAH7* genes were not included in Tables 2 and 3 since the real time PCR results were inconclusive in a number of samples, likely due to low expression levels in those genotypes.

**Table 2:** Relative expression levels of Na^+^ transporter genes in the basal leaf sheaths of rice genotypes IR-44595 and 318 under salinity

Gene	Relative expression level under salinity		
	IR-44595	318	
*OsHKT1;1*	1.60 ± 0.37	1.68 ± 0.28	
*OsHKT1;3*	2.81 ± 0.22	0.45 ± 0.15	**
*OsHKT1;4*	0.20 ± 0.09	0.20 ± 0.09	
*OsHKT1;5*	2.30 ± 0.59	0.37 ± 0.08	*
*OsHKT2;1*	2.89 ± 1.63	1.99 ± 0.79	
*OsHKT2;3*	2.60 ± 0.95	0.10 ± 0.08	
*OsHKT2;4*	0.80 ± 0.15	0.41 ± 0.17	
*OsNHX1*	1.91 ± 0.17	0.36 ± 0.09	**
*OsNHX2*	1.11 ± 0.26	1.24 ± 0.22	
*OsNHX3*	1.02 ± 0.06	0.89 ± 0.08	
*OsNHX5*	0.99 ± 0.04	0.79 ± 0.08	
*OsSOS1*	1.04 ± 0.11	1.06 ± 0.16	

* and ** indicate significant differences between two rice genotypes at *P* < 0.05 and 0.01, respectively

**Table 3:** Relative expression levels of Cl^−^ transporter genes in the apical leaf sheaths of rice genotypes Okshitmayin and WC 4419 under salinity

Gene	Relative expression level under salinity		
	Okshitmayin	WC 4419	
*NPF2;4*	0.63 ± 0.03	0.56 ± 0.10	
*OsCCC1*	1.08 ± 0.12	0.95 ± 0.14	
*OsCLC1*	0.93 ± 0.20	1.01 ± 0.15	
*OsCLC2*	0.82 ± 0.05	0.50 ± 0.06	*
*OsCLC5*	0.32 ± 0.02	0.33 ± 0.05	
*OsCLC6*	0.58 ± 0.10	0.34 ± 0.05	

* indicates a significant difference between two rice genotypes at *P* < 0.05

## Discussion

This study aimed to identify Na^+^ or Cl^−^ transporter genes that are associated with the salt removal ability in leaf sheath of rice and determine how they function in the mechanism of salt removal ability. Therefore, we determined the salt responsiveness of the transcriptional levels of 21 known candidate Na^+^ or Cl^−^ transporter rice genes in the basal or apical parts, respectively, where Na^+^ or Cl^−^ accumulation level was high (Fig. 1). We also determined which known candidate Na^+^ or Cl^−^ transporter genes expressed specifically in the peripheral or central part in leaf sheaths to deduce which genes are related to the function in salt unloading in the peripheral parts or to salt sequestration into fundamental parenchyma cells in the central part.

### Na^+^ transporter genes associated with Na^+^ removal in leaf sheath

The removal of Na^+^ in leaf sheaths was suggested to be regulated by the unloading of Na^+^ from xylem vessels and the sequestration of Na^+^ in the central fundamental parenchyma cells of leaf sheaths [3]. Within HKT transporter protein-encoding genes used in this study, *OsHKT1;1* and *OsHKT1;5* highly increased their expression levels in response to salt stress in the basal part of leaf sheaths (Fig. 2a-g), indicating their potential active functions in Na^+^ transport in the basal part of leaf sheaths upon salt stress. Furthermore, the genes *OsHKT1;3*, *OsHKT1;5*, *OsHKT2;3* and *OsHKT2;4* were highly expressed in the peripheral parts consisting of vasculatures and fundamental parenchyma cells (Fig. 4a-g, Additional file 5). These results indicate that *OsHKT1;5* may be responsible for Na^+^ unloading in the vasculatures from the xylem vessels in leaf sheath under salinity, as a highly selective Na^+^ transporter. *OsHKT1;5* in rice has been characterized to localize on the plasma membrane and be highly expressed in the vasculatures of node and basal stem and leaf sheath of rice exposed to salt stress [16]. It matches with the finding of Kobayashi et al. [16] showing the T-DNA insertion into *OsHKT1;5* decreases the Na^+^ removal ability in leaf sheath. Furthermore, *OsHKT1;5* was also highly expressed in the peripheral parts of leaf sheaths under control conditions (Additional file 3), suggesting the specific role of *OsHKT1;5* in the peripheral region of leaf sheaths. On the other hand, in the leaf sheath, *OsHKT1;1* was expressed in both peripheral and central parts under control and saline conditions (Fig. 4a, Additional files 3, 5, 7) and may function as a Na^+^ transporter in non-specific parts to permeate Na^+^ from apoplastic spaces to the inside of cells under salinity. *OsHKT1;1*, localized on the plasma membrane, is expressed in the vascular tissues of both roots and leaf blades of rice and increased in response to salt stress [10].

The expression levels of all five *OsNHX* genes were also up-regulated under salinity in the basal part of leaf sheath (Fig. 2h-l). Within these *OsNHX* genes, *OsNHX2* and *OsNHX3* were highly expressed in the central part of leaf sheaths under salinity (Fig. 4h-l, Additional file 5). This implies that *OsNHX2* and *OsNHX3* are the responsible genes that sequester Na^+^ ions into the vacuole in the fundamental parenchyma cells in the central part of leaf sheaths during salt stress. The over-accumulation of Na^+^ at the central fundamental parenchyma cells of leaf sheaths is an important process in the salt removal ability in leaf sheath [3]. Therefore, *OsNHX2* and *OsNHX3* are interesting target genes to determine whether Na^+^ sequestration into vacuole in the fundamental parenchyma cells in the central part partly regulate the salt removal ability in leaf sheath. In addition, *OsNHX2* was highly expressed in the central part of leaf sheaths regardless of salt treatment (Fig. 4i, Additional file 3). OsNHX proteins permeate not only Na^+^ but also K^+^ [24]. Fundamental parenchyma cells in the central leaf sheath have been reported to accumulate high concentrations of K^+^ under normal conditions [3]. Therefore, it can be suggested that *OsNHX2* may be involved in K^+^ accumulation in the vacuole of fundamental parenchyma cells in leaf sheath under normal conditions, and it mediates Na^+^ sequestration when excess Na^+^ ions are transported to the leaf sheath. On the other hand, *OsNHX4* and *OsNHX5* expressed highly in the peripheral part of leaf sheath under salinity (Fig. 4k, l), which indicates these two genes function in sequestering Na^+^ into vacuole in peripheral parts (vasculature, epidermis or peripheral parenchyma cells) but not in the central fundamental parenchyma cells of leaf sheaths.

### Cl^−^ transporter genes associated with Cl^−^ removal in leaf sheath

The present results suggested that the removal ability of Cl^−^ in leaf sheath under salt stress conditions is regulated by multiple Cl^−^ transporter genes with diffident mechanisms. The apical part of leaf sheaths showed a significant increase in *OsNPF2;4*, *OsCLC1* and *OsSLAH1* under salinity (Fig. 3). This suggested that these three Cl^−^ transporter genes are involved in the removal ability of Cl^−^ in leaf sheaths under salinity. In addition, *OsNPF2;4* expressed higher in the central part in the comparison to peripheral part of leaf sheaths under saline conditions (Fig. 5). *NPF2;4*, localized on the plasma membrane, have been reported to work in loading Cl^−^ into the xylem vessel in the root stele of *Arabidopsis* [35]. In rice, *OsNPF2;4* has been reported as a low-affinity nitrate transporter and is involved in long-distance transportation of nitrate from roots to shoots [41], although the physiological functions of *OsNPF2;4* as a chloride transporter in rice have been not studied so far. It is suggested that *OsNPF2;4* may function in Cl^−^ loading into fundamental parenchyma cells in the central part of leaf sheath under salinity, although further investigations are required to confirm the involvement of NPF2;4 in the removal of Cl^−^ in leaf sheath of rice. On the other hand, *OsSLAH1*, a plasma membrane-type Cl^−^ transporter [36] expressed higher in the peripheral part compared with central part under both normal and salt-treated conditions (Fig. 5g, Additional file 4), suggesting the specific function of *OsSLAH1* in the peripheral part of leaf sheaths. In *Arabidopsis*, homologous genes *AtSLAH1* and *AtSLAH3* co-express in root stele cells and function in Cl^−^ loading into xylem as a Cl^−^ efflux transporter [42]. It was suspected that *OsSLAH1* may function in Cl^−^ transportation from peripheral part to central part via the function of Cl^−^ efflux, although it has to be needed to investigate the physiological function of *OsSLAH1* in the salt removal ability in leaf sheath.

*OsCLC1*, mainly localized in the tonoplast, is known as a ﻿rice chloride channel gene to mediate compartmentalizing Cl^−^ into the vacuole under salt stress [39]. *OsCLC1* expressed relatively highly in the central part compared with peripheral part (Fig. 5), which indicated that *OsCLC1* may work in sequestering Cl^−^ into the vacuole of fundamental parenchyma cells in the central parts of leaf sheaths. *OsCLC2* and *OsCLC6* also expressed highly in the central parts (Fig. 5, Additional file 6), whereas these genes may not be involved in the Cl^−^ removal ability in leaf sheath under salinity since these genes were down-regulated by salinity.

### Genotypic comparison of the expression levels of Na^+^ and Cl^−^ transporter genes under salinity

We determined causal genes of the genotypic difference regarding Na^+^ or Cl^−^ removal ability in leaf sheaths. Under salinity, IR-44595 with superior Na^+^ removal ability in the leaf sheaths showed higher expression levels of *OsHKT1;3*, *OsHKT1;5* and *OsNHX1* genes in comparison to the cultivar 318 with the inferior ability (Table 2). This result indicates that high expression levels of those genes may be associated with higher Na^+^ removal ability in leaf sheaths. It is also in agreement with the previous finding where the knockout of the *OsHKT1;5* gene decreases the ability of Na^+^ removal ability in leaf sheath [16]. So far, there has been no report regarding the involvement of *OsNHX1* in the Na^+^ removal ability in leaf sheath. *OsNHX1* seemed to be expressed relatively higher in the central parenchyma cells in leaf sheath (Fig. 4), indicating the involvement of the gene in Na^+^ sequestration in the cells. These results may indicate the Na^+^ sequestration in the fundamental parenchyma cells in the central part of leaf sheath can also quantitatively affect the ability in the Na^+^ removal ability in leaf sheath, as well as the Na^+^ unloading activity in the peripheral part regulated by *OsHKT1;5*. Further studies are necessary to confirm this hypothesis by determining the effect of altered expression level of *OsNHX1* gene on the Na^+^ removal ability in the leaf sheath. Wangsawang et al. [43] reported that, in a salt-tolerant japonica rice variety Ouukan 383, highly-induced expression of *OsHKT1;4* possibly corresponds to the high Na^+^ accumulation in the leaf sheath. It implies the causal genes of genotypic variation may be dependent on genotypes.

In contrast, regarding genotypic difference in Cl^−^ removal ability in leaf sheaths, a higher expression level of *OsCLC2* was associated with higher Cl^−^ removal ability in Okshitmayin in comparison to WC 4419 (Table 3). *OsCLC2* may be involved in Cl^−^ sequestration by preferentially expressing in the central fundamental parenchyma cells in leaf sheaths (Fig. 5), indicating that Cl^−^ sequestration activity possibly affects the Cl^−^ removal ability in the leaf sheath. *OsCLC2* gene encodes a vacuolar voltage-gated chloride channel in rice and is preferentially expressed in leaf sheaths than leaf blades [39], although direct evidence of the involvement of *OsCLC2* in salt tolerance, especially Cl^−^ removal in leaf sheath, has not reported so far. In Arabidopsis plants, *AtCLC* genes are involved in salt tolerance [44, 45]. Also, in wild soybean BB52, the *GsCLC-*_*C2*_ gene expresses in the roots and functions in Cl^−^ sequestration in roots, which contributes to lowering Cl^−^ transportation from roots to shoots [46]. Further studies are necessary to determine the physiological role of *OsCLC2* in leaf sheath using *OsCLC2*-knockout or overexpressed rice mutants and validate if the difference in *OsCLC2* expression levels causes the genotypic variation in the Cl^−^ removal ability in rice leaf sheaths.

## Conclusion

The present study indicates that a number of known candidate Na^+^ or Cl^−^ transporter genes respond to salinity in the leaf sheath. Furthermore, it demonstrated the tissue-specific expression of some Na^+^ or Cl^−^ transporter genes indicating the involvement of different genes in the salt unloading in the peripheral part and salt sequestration in the central part of leaf sheath. Concerning the removal of Na^+^ in the leaf sheath, *OsHKT1;5* probably functions in Na^+^ unloading from xylem vessels during salt stress. Na^+^ sequestration in the fundamental parenchyma cells at the central part of leaf sheath may be associated with *OsNHX2* and *OsNHX3* under saline conditions. The removal ability of Cl^−^ in the leaf sheaths may be regulated by several Cl^−^ transporters such as *OsNPF2;4*, *OsCLC1* and *OsSLAH1* under salt stress conditions. Furthermore, *OsCLC1* is strongly suggested to play a role in accumulating Cl^−^ in the fundamental parenchyma cells at the central region of internal leaf sheaths under saline conditions. Also, some genes were identified as candidates whose expression levels were associated with the genotypic variation of salt removal ability in the leaf sheath. Further investigation is necessary to validate the physiological function of each gene in the mechanism of salt removal in leaf sheath by using gene knockout or gene overexpression techniques. Additionally, it is necessary to investigate the correlation between salt tolerant traits and candidate gene expression levels moving towards the possibility of the utility of these genes in breeding to produce new superior salt tolerant rice varieties.

## Methods

### Plant materials and growth conditions

The salt-tolerant rice genotype, FL 478, was used in this study. The FL 478 seeds were kindly provided by Genebank at the International Rice Research Institute (IRRI), the Philippines, and propagated at Nagoya University, Japan. The growth conditions of rice seedling were described in Neang et al. [3]. Plants were hydroponically grown for 2 weeks in Yoshida solution [47], then 50 mM NaCl was added to the hydroponic solution for 2 days followed by 100 mM NaCl for 1 day (in total 3 days).

### Sample preparation

The fifth leaves were cut into 4 total parts; sheaths were cut into 3 parts with same length, the 4th part consisted of the leaf blade (Sheath1, Sheath2, Sheath3, Blade). Sheath1, Sheath2 and Sheath3 refer to basal, middle and upper parts of leaf sheaths, and Blade refers to whole leaf blades. The four parts were used for the measurement of Na^+^ and Cl^−^ concentrations. The Sheath1 (basal leaf sheath) and Sheath3 (apical leaf sheath) were used for gene expression analysis. In addition, the middle part of fifth leaf sheath was used to make cross sections. Each cross section first was divided into upper and lower part, with upper parts separated into central and peripheral parts using a razor blade under a stereo microscope (Olympus SZ61) (Additional file 9). The lower parts of cross section were not used because it was difficult to separate into central and peripheral parts. The central part of cross-sectioned leaf sheath consisted of mostly fundamental parenchyma cells, and peripheral part consisted of fundamental parenchyma cells, epidermis and vasculatures. After harvesting, samples for RNA extraction were immediately put into liquid N_2_, then stored at -80 °C if necessary. Samples for Na^+^ and Cl^−^ measurement were dried at 70 °C for more than 72 h.

### ﻿Measurement of Na^+^ and Cl^−^ concentrations

Na^+^ and Cl^−^ concentrations were measured as described in Neang et al. [3].

### Gene expression analysis

Total RNA was isolated from basal, middle, and apical parts of leaf sheath, central and peripheral parts of cross-sectioned leaf sheath using a RNeasy Plant Mini Kit (QIAGEN, Hilden, Germany). The extracted RNA of each sample was used for cDNA synthesis with an oligo (dT)^15^ primer (Takara Bio, Shiga, Japan) using TAKARA PrimeScript reverse transcriptase (Takara Bio, Shiga, Japan). Then, Real-Time PCR analysis was performed with SYBR Premix Ex Taq II (Takara Bio, Shiga, japan) using LightCycler 96 (Roche Diagnostics,Risch-Rotkreuz, Switzerland) instruments. Gene-specific primer sets are listed in Additional file 10. The relative transcript level of each gene was calculated using the 2^−∆∆CT^ method [48] using *OsActin1* as the control gene.

### RNA-seq analysis

RNA samples extracted from the central and peripheral parts of the leaf sheath of FL 478 as described above were also used for RNA-seq analysis to validate the data of Real-Time PCR analyses. The quality check of RNA and RNA-seq analysis was conducted as described in Neang et al. [40]. The data has been deposited into DDBJ Sequence Read Archive (accession no. DRA009377). The relative transcript level of each gene was calculated by dividing the reads per kilobase per million reads (RPKM) of each gene by RPKM of *OsActin1* as the control gene.

### Genotypic comparison of Na^+^ and Cl^−^ transporter gene expressions

For the comparison of the expression levels of Na^+^ and Cl^−^ transporter genes between genotypes that show high and low ability of Na^+^ or Cl^−^ removal in the leaf sheath, four rice genotypes, IR-44595 (*indica*, high Na^+^ removal in leaf sheath, IRGC accession number 117755), 318 (*tropical japonica*, low Na^+^ removal in leaf sheath, IRGC accession number 117629), Okshitmayin (*admix*, high Cl^−^ removal in leaf sheath, IRGC accession number 117827) and WC 4419 (*tropical japonica*, low Cl^−^ removal in leaf sheath, IRGC accession number 117626) screened from 296 rice genotypes [40], were used. These seeds were kindly provided by Genebank at IRRI. They were cultivated and salinized as described above. Fifth leaves were harvested and cut into four parts, basal, middle, apical leaf sheaths and leaf blades as described above. Na^+^ and Cl^−^ concentrations in each part were measured and the parts that showed the highest concentration of Na^+^ or Cl^−^ were used for RNA extraction. Then the extracted RNA was used for determining the expression levels of Na^+^ or Cl^−^ transporter genes by Real-Time PCR analysis as described above.

### Statistical analysis

Three replicates were used per treatment. Data were statistically analyzed using analysis of variance (ANOVA), *t*-test and Tukey’s multiple comparison test running by R software. Significant differences were analyszed based on *P < 0.05* and *0.01*.

## Supplementary Information

**Additional file 1 **Relative expression levels of Na^+^ transporter genes in the middle and apical parts of leaf sheaths under control or treatment conditions with 100 mM NaCl. Data are mean of three replications ± the standard error. * indicates significant difference at *P* < 0.05 between conditions.

**Additional file 2 **Relative expression levels of Cl^−^ transporter genes in the basal and middle parts of leaf sheath under control or treatment conditions with 100 mM NaCl. Data are mean of three replications ± ﻿the standard error. * indicates significant difference at *P* < 0.05 between conditions.

**Additional file 3 **Relative expression levels of Na^+^ transporter genes in the central and peripheral parts of leaf sheath under control conditions. Data are mean of three replications ± the standard error. * indicates significant difference at *P* < 0.05 between two parts.

**Additional file 4 **Relative expression levels of Cl^−^ transporter genes in the central and peripheral parts of leaf sheath under control conditions. Data are mean of three replications ± the standard error. * indicates significant difference at *P* < 0.05 between two parts.

**Additional file 5 **Validations of Na^+^ transporter genes using RNA-seq analysis in the central and peripheral parts of leaf sheath under treatment conditions with 100 mM NaCl. Data are mean of three replications ± the standard error. * indicates significant difference at *P* < 0.05 between two parts.

**Additional file 6 **Validations of Cl^−^ transporter genes using RNA-seq analysis in the central and peripheral parts of leaf sheath under treatment conditions with 100 mM NaCl. Data are mean of three replications ± the standard error. * indicates significant difference at *P* < 0.05 between two parts.

**Additional file 7 **Validations of Na^+^ transporter genes using RNA-seq analysis in the central and peripheral parts of leaf sheath under control conditions. Data are mean of three replications ± the standard error. * indicates significant difference at *P* < 0.05 between two parts.

**Additional file 8 **Validations of Cl^−^ transporter genes using RNA-seq analysis in the central and peripheral parts of leaf sheath under control conditions. Data are mean of three replications ± the standard error. * indicates significant difference at *P* < 0.05 between two parts.

**Additional file 9.** Sample preparation. a) a cross section of leaf sheath, b) Separation into upper and lower parts of cross-sectioned leaf sheath, c) Separation into peripheral and central parts, d) Central part after separating peripheral part.

**Additional file 10.** All primers used for RT-PCR.

## Data Availability

All data analyzed or analyzed during this study are included in this published article and its additional files. The sequence data generated and/or analysed during the current study are available in the DDBJ Sequence Read Archive repository via accession number DRA009377 (ftp://ftp.ddbj.nig.ac.jp/ddbj_database/dra/fastq/DRA009/DRA009377/). Plant materials used throughout this work are available from Genebank at IRRI, the Philippines.

## Abbreviations

ANOVA: Analysis of variance
CCC1: Cation-chloride transporter 1
CLC: Chloride channel
HKT1: Group 1 high-affinity K^+^ transporter
IRRI: International rice research institute
NHX: Na^+^/H^+^ exchanger
NPF2.4: Nitrate transporter 1/peptide transporter 2.4
RPKM: Reads per kilobase per million reads
SOS1: Salt-overly-sensitive 1
