# Supplementary material for: Tissue-specific expression analysis of Na+ and Cl− transporter genes associated with salt removal ability in rice leaf sheath
Source: BMC Plant Biol. 2020 Nov 3;20:502. doi: 10.1186/s12870-020-02718-4 (PMC7607675; doi:10.1186/s12870-020-02718-4)
Supplement: Supplementary file 1 — Additional file 1 Relative expression levels of Na+ transporter genes in the middle and apical parts of leaf sheaths under control or treatment conditions with 100 mM NaCl. Data are mean of three replications ± the standard error. * indicates significant difference at P < 0.05 between conditions. [file 12870_2020_2718_MOESM1_ESM.pptx]

## Slide 1
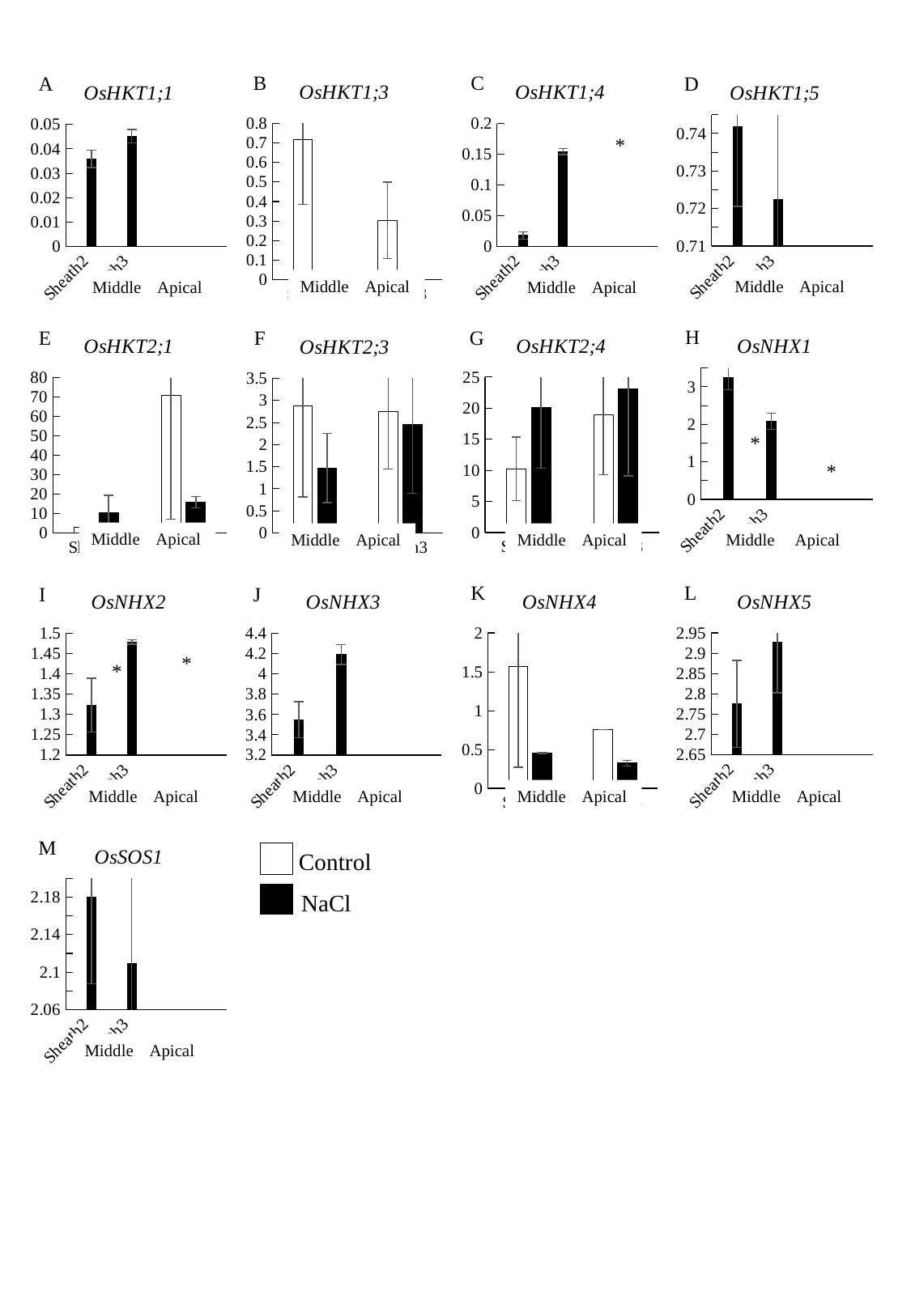

B
### Chart: OsHKT1;3
| Category | | |
|---|---|---|
| Sheath2 | 0.7153004278196273 | 0.0046451285021843315 |
| Sheath3 | 0.3038751473723946 | 0.014681790549065042 |C
### Chart: OsHKT1;4
| Category | | |
|---|---|---|
| Sheath2 | 0.01612 | 0.018056666666666665 |
| Sheath3 | 0.02934 | 0.15469 |A
### Chart: OsHKT1;5
| Category | | |
|---|---|---|
| Sheath2 | 0.5552333333333334 | 0.7417666666666666 |
| Sheath3 | 0.43283333333333335 | 0.7223 |
### Chart: OsHKT1;1
| Category | | |
|---|---|---|
| Sheath2 | 0.04458 | 0.03593 |
| Sheath3 | 0.06076 | 0.04520666666666667 |D
*
Middle Apical
Middle Apical
Middle Apical
Middle Apical
### Chart: OsHKT2;4
| Category | | |
|---|---|---|
| Sheath2 | 10.233048000547335 | 20.06649930918073 |
| Sheath3 | 18.959662444970927 | 23.05742401538242 |H
### Chart: OsNHX1
| Category | | |
|---|---|---|
| Sheath2 | 6.468 | 3.2406666666666673 |
| Sheath3 | 4.9126666666666665 | 2.076 |
### Chart: OsHKT2;1
| Category | | |
|---|---|---|
| Sheath2 | 2.981969229861765 | 10.608867261118942 |
| Sheath3 | 70.90020391257029 | 15.832759908266382 |E
G
F
### Chart: OsHKT2;3
| Category | | |
|---|---|---|
| Sheath2 | 2.875278883203775 | 1.471669765768466 |
| Sheath3 | 2.7578491431287255 | 2.464132131575753 |*
*
Middle Apical
Middle Apical
Middle Apical
Middle Apical
K
L
### Chart: OsNHX4
| Category | | |
|---|---|---|
| Sheath2 | 1.569797105831588 | 0.45695963112530114 |
| Sheath3 | 0.7578582832551979 | 0.3263325876532005 |
### Chart: OsNHX5
| Category | | |
|---|---|---|
| Sheath2 | 2.3523333333333336 | 2.775 |
| Sheath3 | 3.0239999999999996 | 2.9273333333333333 |
### Chart: OsNHX2
| Category | | |
|---|---|---|
| Sheath2 | 0.9962333333333334 | 1.3226666666666667 |
| Sheath3 | 1.2096666666666667 | 1.4783333333333335 |
### Chart: OsNHX3
| Category | | |
|---|---|---|
| Sheath2 | 4.098333333333334 | 3.5483333333333333 |
| Sheath3 | 4.8116666666666665 | 4.191333333333334 |I
J
*
*
Middle Apical
Middle Apical
Middle Apical
Middle Apical
M
### Chart: OsSOS1
| Category | | |
|---|---|---|
| Sheath2 | 2.324666666666667 | 2.180333333333333 |
| Sheath3 | 1.852 | 2.1093333333333337 |Control
NaCl
Middle Apical
