# Supplementary material for: Tissue-specific expression analysis of Na+ and Cl− transporter genes associated with salt removal ability in rice leaf sheath
Source: BMC Plant Biol. 2020 Nov 3;20:502. doi: 10.1186/s12870-020-02718-4 (PMC7607675; doi:10.1186/s12870-020-02718-4)
Supplement: Supplementary file 2 — Additional file 2 Relative expression levels of Cl− transporter genes in the basal and middle parts of leaf sheath under control or treatment conditions with 100 mM NaCl. Data are mean of three replications ± ﻿the standard error. * indicates significant difference at P < 0.05 between conditions. [file 12870_2020_2718_MOESM2_ESM.pptx]

## Slide 1
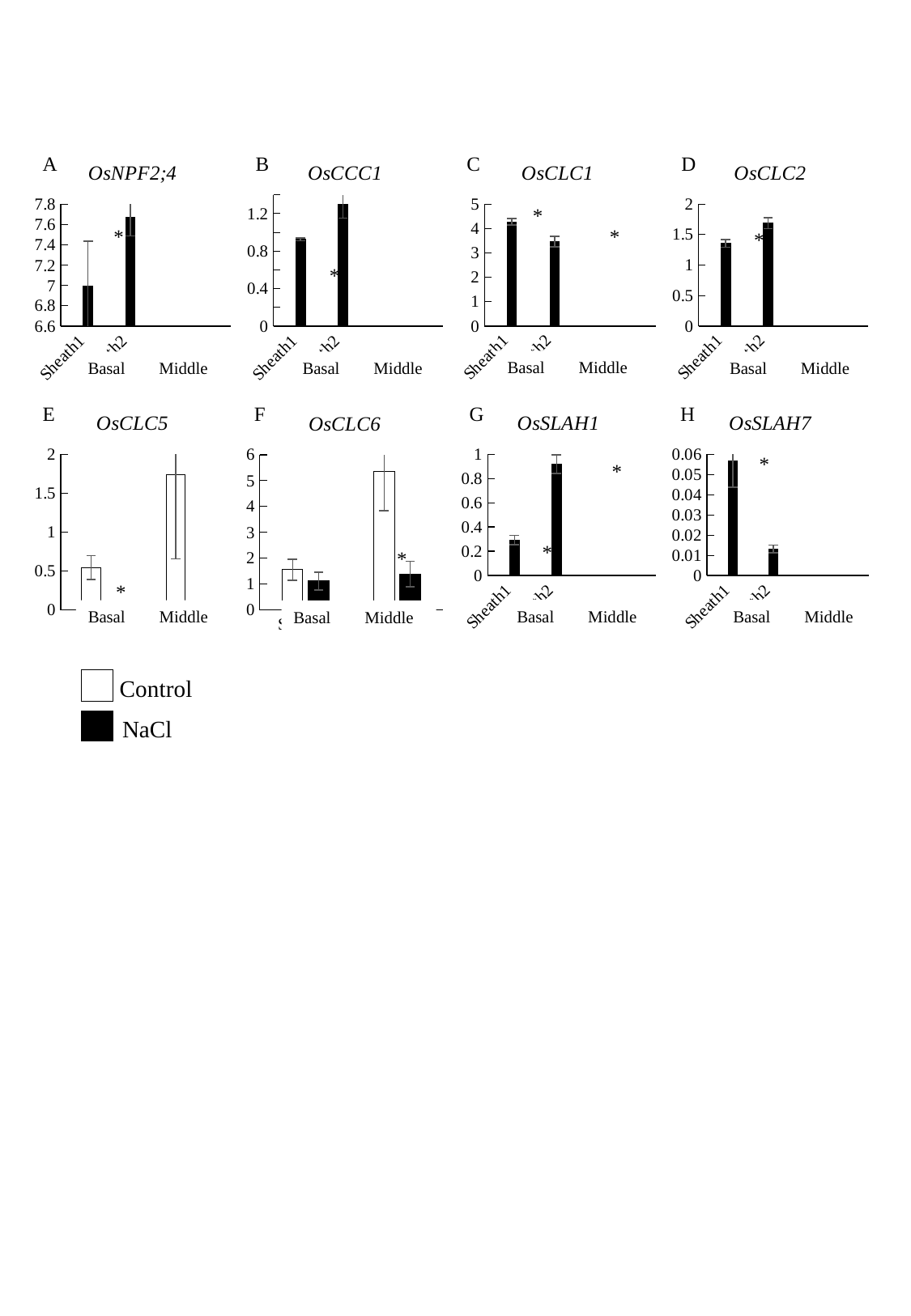

D
A
B
C
### Chart: OsCLC2
| Category | | |
|---|---|---|
| Sheath1 | 0.23183333333333334 | 1.3539999999999999 |
| Sheath2 | 1.5206666666666668 | 1.689 |
### Chart: OsCLC1
| Category | | |
|---|---|---|
| Sheath1 | 0.21486666666666668 | 4.2749999999999995 |
| Sheath2 | 1.6273333333333333 | 3.4563333333333333 |
### Chart: OsNPF2;4
| Category | | |
|---|---|---|
| Sheath1 | 1.7990000000000002 | 6.991333333333333 |
| Sheath2 | 8.351333333333335 | 7.669333333333334 |
### Chart: OsCCC1
| Category | | |
|---|---|---|
| Sheath1 | 0.36190000000000005 | 0.9256333333333333 |
| Sheath2 | 1.0563333333333336 | 1.2953333333333334 |*
*
*
*
*
Basal Middle
Basal Middle
Basal Middle
Basal Middle
G
H
### Chart: OsCLC5
| Category | | |
|---|---|---|
| Sheath1 | 0.5446395765507109 | 0.018400043536688674 |
| Sheath2 | 1.7332604840294683 | 0.02306014506578013 |E
F
### Chart: OsSLAH1
| Category | | |
|---|---|---|
| Sheath1 | 0.07321 | 0.29216666666666663 |
| Sheath2 | 0.7084666666666667 | 0.9178333333333333 |
### Chart: OsSLAH7
| Category | | |
|---|---|---|
| Sheath1 | 0.012409333333333333 | 0.056693333333333325 |
| Sheath2 | 0.05058666666666667 | 0.01319 |
### Chart: OsCLC6
| Category | | |
|---|---|---|
| Sheath1 | 1.5611740013693873 | 1.121563531727972 |
| Sheath2 | 5.342670615920102 | 1.3900037796080074 |*
*
*
*
*
Basal Middle
Basal Middle
Basal Middle
Basal Middle
Control
NaCl
