# Supplementary material for: Tissue-specific expression analysis of Na+ and Cl− transporter genes associated with salt removal ability in rice leaf sheath
Source: BMC Plant Biol. 2020 Nov 3;20:502. doi: 10.1186/s12870-020-02718-4 (PMC7607675; doi:10.1186/s12870-020-02718-4)
Supplement: Supplementary file 3 — Additional file 3 Relative expression levels of Na+ transporter genes in the central and peripheral parts of leaf sheath under control conditions. Data are mean of three replications ± the standard error. * indicates significant difference at P < 0.05 between two parts. [file 12870_2020_2718_MOESM3_ESM.pptx]

## Slide 1
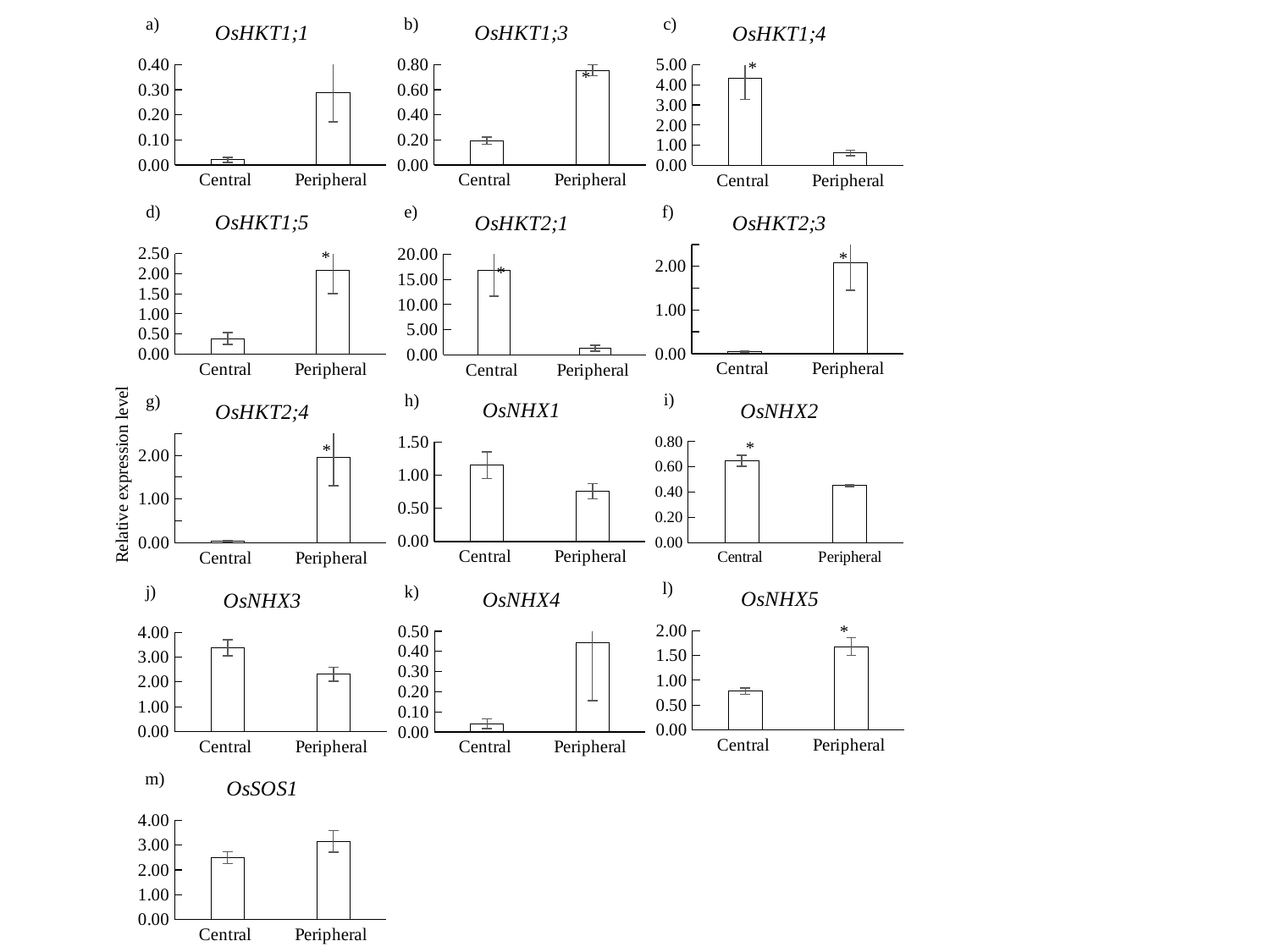

### Chart: OsHKT1;1
| Category | |
|---|---|
| Central | 0.020356701153387773 |
| Peripheral | 0.2874988809722805 |
### Chart: OsHKT1;3
| Category | |
|---|---|
| Central | 0.19354500016726917 |
| Peripheral | 0.7551599975978274 |
### Chart: OsHKT1;4
| Category | |
|---|---|
| Central | 4.349114904127831 |
| Peripheral | 0.6118212563275826 |c)
a)
b)
*
*
d)
### Chart: OsHKT1;5
| Category | |
|---|---|
| Central | 0.38605680444946916 |
| Peripheral | 2.080563399998471 |f)
e)
### Chart: OsHKT2;1
| Category | |
|---|---|
| Central | 16.721934198736367 |
| Peripheral | 1.314115284282259 |
### Chart: OsHKT2;3
| Category | |
|---|---|
| Central | 0.05321536394095417 |
| Peripheral | 2.0754872009894254 |*
*
*
i)
h)
### Chart: OsNHX1
| Category | |
|---|---|
| Central | 1.1512934185031098 |
| Peripheral | 0.7563475333856001 |
### Chart: OsNHX2
| Category | |
|---|---|
| Central | 0.6473 |
| Peripheral | 0.4518333333333333 |g)
### Chart: OsHKT2;4
| Category | |
|---|---|
| Central | 0.03591394137345081 |
| Peripheral | 1.9464941106639404 |*
*
Relative expression level
l)
### Chart: OsNHX5
| Category | |
|---|---|
| Central | 0.7777034058622042 |
| Peripheral | 1.676849261155323 |
### Chart: OsNHX4
| Category | |
|---|---|
| Central | 0.039933600618577335 |
| Peripheral | 0.4414177703780731 |
### Chart: OsNHX3
| Category | |
|---|---|
| Central | 3.37898120124482 |
| Peripheral | 2.3053925082237865 |j)
k)
*
m)
### Chart: OsSOS1
| Category | |
|---|---|
| Central | 2.491396723695486 |
| Peripheral | 3.1532460208614546 |
