# Supplementary material for: Tissue-specific expression analysis of Na+ and Cl− transporter genes associated with salt removal ability in rice leaf sheath
Source: BMC Plant Biol. 2020 Nov 3;20:502. doi: 10.1186/s12870-020-02718-4 (PMC7607675; doi:10.1186/s12870-020-02718-4)
Supplement: Supplementary file 4 — Additional file 4 Relative expression levels of Cl− transporter genes in the central and peripheral parts of leaf sheath under control conditions. Data are mean of three replications ± the standard error. * indicates significant difference at P < 0.05 between two parts. [file 12870_2020_2718_MOESM4_ESM.pptx]

## Slide 1
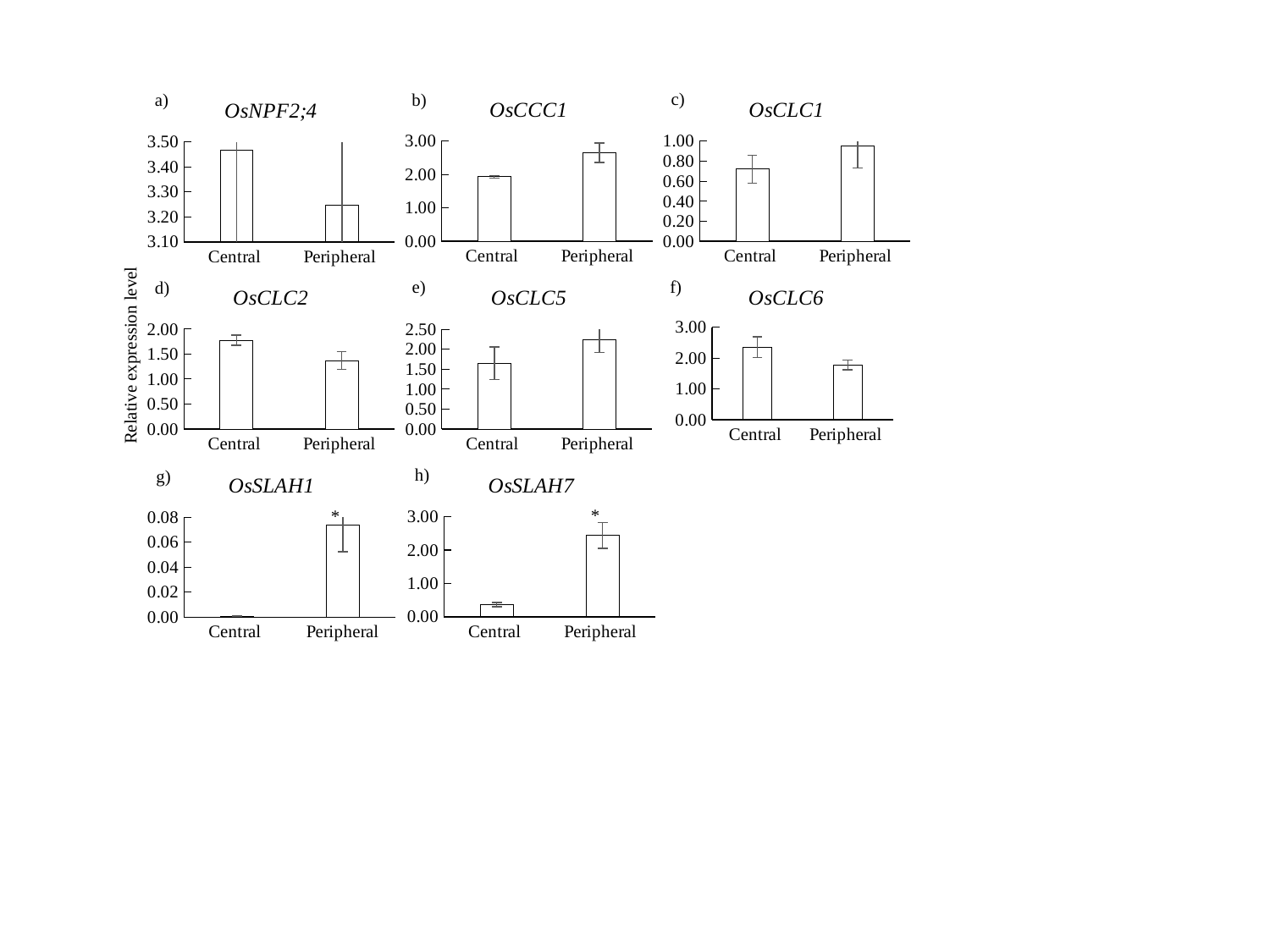

c)
b)
### Chart: OsCCC1
| Category | |
|---|---|
| Central | 1.9327138956719379 |
| Peripheral | 2.648779897026445 |a)
### Chart: OsCLC1
| Category | |
|---|---|
| Central | 0.7207254072604746 |
| Peripheral | 0.9496975461068734 |
### Chart: OsNPF2;4
| Category | |
|---|---|
| Central | 3.467247143457444 |
| Peripheral | 3.2450523195285714 |f)
e)
### Chart: OsCLC2
| Category | |
|---|---|
| Central | 1.7756645307369208 |
| Peripheral | 1.3692823797647062 |d)
### Chart: OsCLC5
| Category | |
|---|---|
| Central | 1.6473097792037175 |
| Peripheral | 2.2254771740421426 |
### Chart: OsCLC6
| Category | |
|---|---|
| Central | 2.3566137924569346 |
| Peripheral | 1.7793691169220687 |Relative expression level
h)
### Chart: OsSLAH7
| Category | |
|---|---|
| Central | 0.35888635368011707 |
| Peripheral | 2.4395901818117114 |
### Chart: OsSLAH1
| Category | |
|---|---|
| Central | 0.0007879333333333334 |
| Peripheral | 0.07358666666666668 |g)
*
*
