# Supplementary material for: Tissue-specific expression analysis of Na+ and Cl− transporter genes associated with salt removal ability in rice leaf sheath
Source: BMC Plant Biol. 2020 Nov 3;20:502. doi: 10.1186/s12870-020-02718-4 (PMC7607675; doi:10.1186/s12870-020-02718-4)
Supplement: Supplementary file 5 — Additional file 5 Validations of Na+ transporter genes using RNA-seq analysis in the central and peripheral parts of leaf sheath under treatment conditions with 100 mM NaCl. Data are mean of three replications ± the standard error. * indicates significant difference at P < 0.05 between two parts. [file 12870_2020_2718_MOESM5_ESM.pptx]

## Slide 1
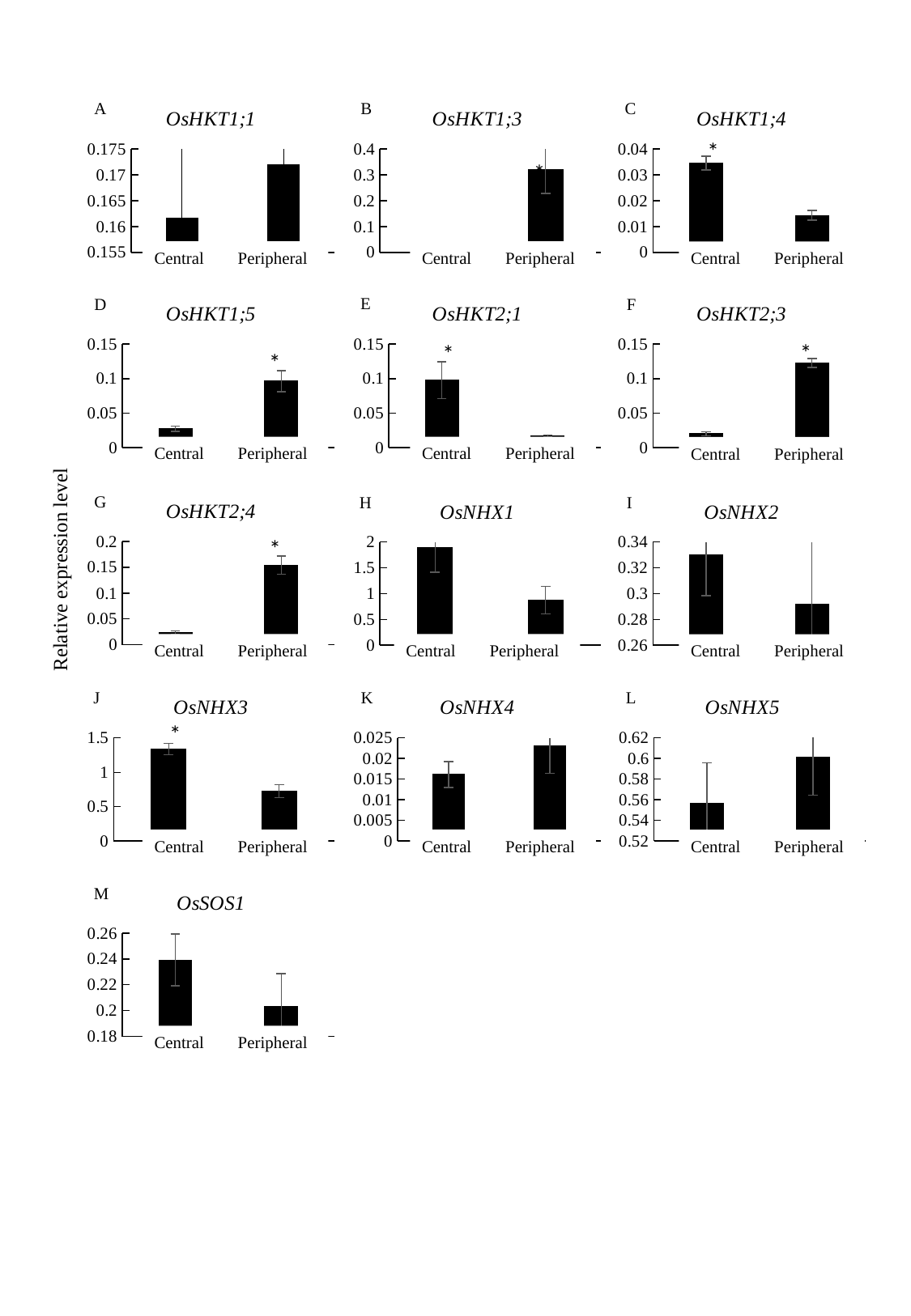

C
### Chart: OsHKT1;1
| Category | |
|---|---|
### Chart: OsHKT1;3
| Category | |
|---|---|B
### Chart: OsHKT1;4
| Category | |
|---|---|A
*
*
Central Peripheral
Central Peripheral
Central Peripheral
### Chart: OsHKT1;5
| Category | |
|---|---|E
### Chart: OsHKT2;1
| Category | |
|---|---|
### Chart: OsHKT2;3
| Category | |
|---|---|F
D
*
*
*
Central Peripheral
Central Peripheral
Central Peripheral
### Chart: OsHKT2;4
| Category | |
|---|---|
### Chart: OsNHX2
| Category | |
|---|---|
### Chart: OsNHX1
| Category | |
|---|---|G
I
H
*
Relative expression level
Central Peripheral
Central Peripheral
Central Peripheral
L
K
### Chart: OsNHX5
| Category | |
|---|---|J
### Chart: OsNHX3
| Category | |
|---|---|
### Chart: OsNHX4
| Category | |
|---|---|*
Central Peripheral
Central Peripheral
Central Peripheral
### Chart: OsSOS1
| Category | |
|---|---|M
Central Peripheral
