# Supplementary material for: Tissue-specific expression analysis of Na+ and Cl− transporter genes associated with salt removal ability in rice leaf sheath
Source: BMC Plant Biol. 2020 Nov 3;20:502. doi: 10.1186/s12870-020-02718-4 (PMC7607675; doi:10.1186/s12870-020-02718-4)
Supplement: Supplementary file 7 — Additional file 7 Validations of Na+ transporter genes using RNA-seq analysis in the central and peripheral parts of leaf sheath under control conditions. Data are mean of three replications ± the standard error. * indicates significant difference at P < 0.05 between two parts. [file 12870_2020_2718_MOESM7_ESM.pptx]

## Slide 1
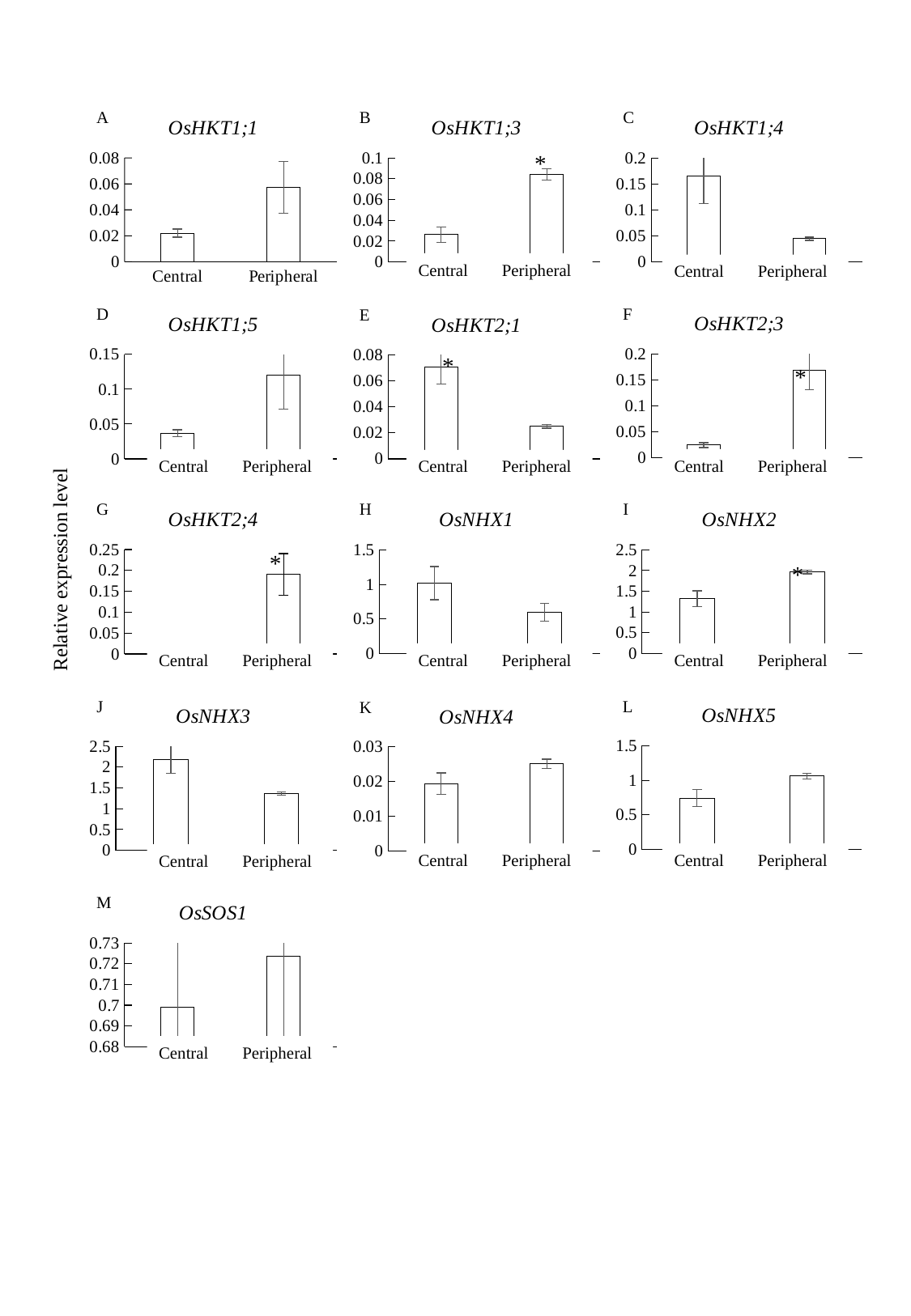

B
### Chart: OsHKT1;1
| Category | |
|---|---|
| Central | 0.02202591949972718 |
| Peripheral | 0.057257402921427204 |A
### Chart: OsHKT1;3
| Category | |
|---|---|C
### Chart: OsHKT1;4
| Category | |
|---|---|*
Central Peripheral
Central Peripheral
D
F
### Chart: OsHKT2;3
| Category | |
|---|---|
### Chart: OsHKT1;5
| Category | |
|---|---|E
### Chart: OsHKT2;1
| Category | |
|---|---|*
*
Central Peripheral
Central Peripheral
Central Peripheral
I
H
### Chart: OsHKT2;4
| Category | |
|---|---|G
### Chart: OsNHX1
| Category | |
|---|---|
### Chart: OsNHX2
| Category | |
|---|---|*
Relative expression level
*
Central Peripheral
Central Peripheral
Central Peripheral
### Chart: OsNHX5
| Category | |
|---|---|
### Chart: OsNHX3
| Category | |
|---|---|J
### Chart: OsNHX4
| Category | |
|---|---|L
K
Central Peripheral
Central Peripheral
Central Peripheral
M
### Chart: OsSOS1
| Category | |
|---|---|Central Peripheral
