# Supplementary material for: Tissue-specific expression analysis of Na+ and Cl− transporter genes associated with salt removal ability in rice leaf sheath
Source: BMC Plant Biol. 2020 Nov 3;20:502. doi: 10.1186/s12870-020-02718-4 (PMC7607675; doi:10.1186/s12870-020-02718-4)
Supplement: Supplementary file 8 — Additional file 8 Validations of Cl− transporter genes using RNA-seq analysis in the central and peripheral parts of leaf sheath under control conditions. Data are mean of three replications ± the standard error. * indicates significant difference at P < 0.05 between two parts. [file 12870_2020_2718_MOESM8_ESM.pptx]

## Slide 1
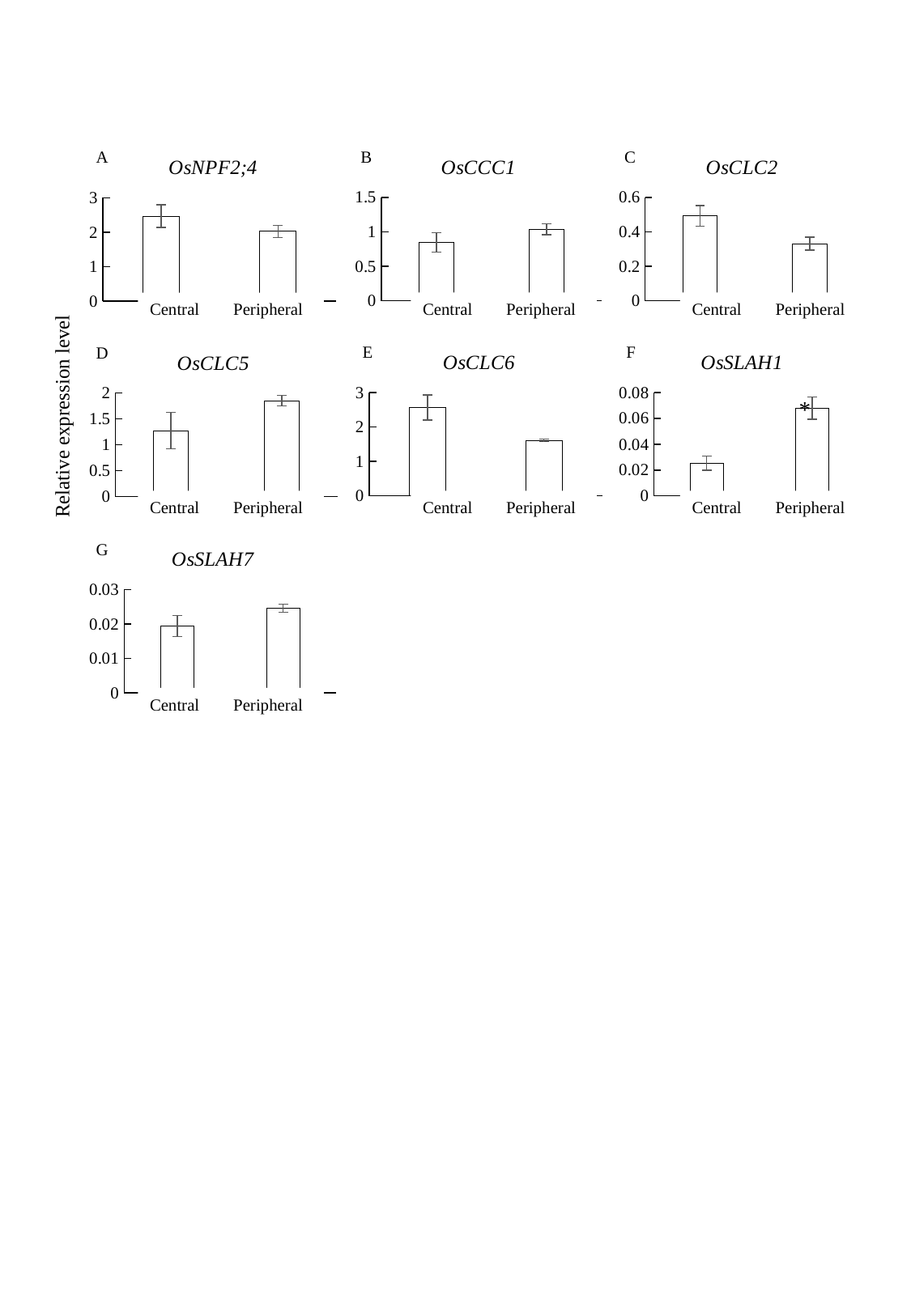

### Chart: OsCCC1
| Category | |
|---|---|C
### Chart: OsCLC2
| Category | |
|---|---|A
B
### Chart: OsNPF2;4
| Category | |
|---|---|Central Peripheral
Central Peripheral
Central Peripheral
E
### Chart: OsCLC6
| Category | |
|---|---|
### Chart: OsSLAH1
| Category | |
|---|---|F
### Chart: OsCLC5
| Category | |
|---|---|D
*
Relative expression level
Central Peripheral
Central Peripheral
Central Peripheral
### Chart: OsSLAH7
| Category | |
|---|---|G
Central Peripheral
