# Supplementary figures and images for: Tissue-specific expression analysis of Na+ and Cl− transporter genes associated with salt removal ability in rice leaf sheath
Source: BMC Plant Biol. 2020 Nov 3;20:502. doi: 10.1186/s12870-020-02718-4 (PMC7607675; doi:10.1186/s12870-020-02718-4)

## Slide 1
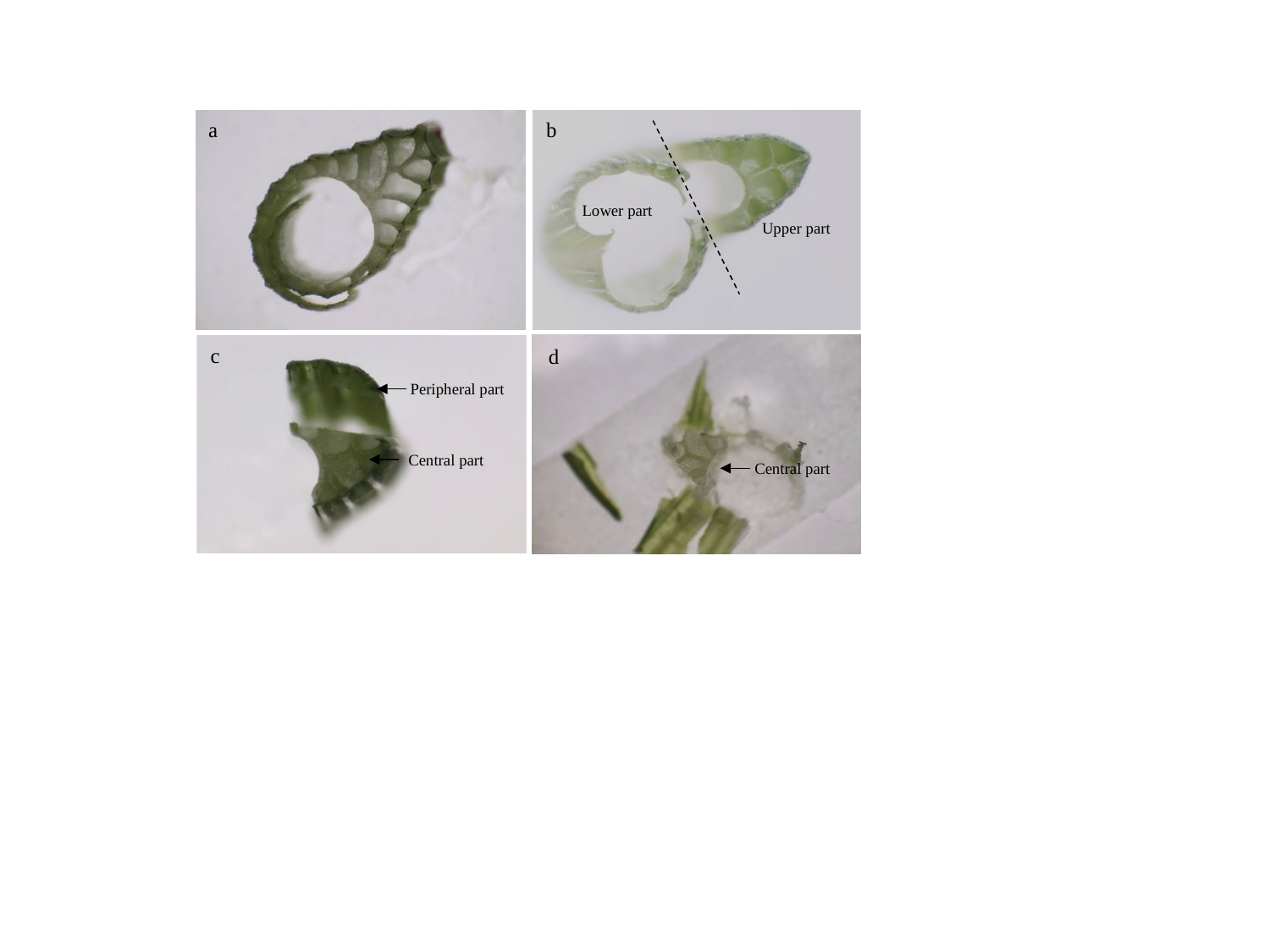

b
a
Lower part
Upper part
c
d
Peripheral part
Central part
Central part

Supplement: Supplementary file 9 — Additional file 9. Sample preparation. a) a cross section of leaf sheath, b) Separation into upper and lower parts of cross-sectioned leaf sheath, c) Separation into peripheral and central parts, d) Central part after separating peripheral part. [file 12870_2020_2718_MOESM9_ESM.pptx]
